# Parastomal Hernia Repair with Intraperitoneal Mesh

**DOI:** 10.1155/2017/8597463

**Published:** 2017-10-24

**Authors:** Pia Näsvall, Jörgen Rutegård, Michael Dahlberg, Ulf Gunnarsson, Karin Strigård

**Affiliations:** ^1^Department of Surgery, Sunderby Hospital, Umeå University, Luleå, Sweden; ^2^Department of Surgery and Perioperative Sciences, Umeå University, Umeå, Sweden

## Abstract

**Purpose:**

Parastomal hernia is a common complication following a stoma and may cause leakage or incarceration. No optimal treatment has been established, and existing methods using mesh repair are associated with high recurrence rates and a considerable risk for short- and long-term complications including death. A double-layer intraperitoneal on-lay mesh (IPOM), the Parastomal Hernia Patch (BARD™), consisting of ePTFE and polypropylene, has been developed and tailored to avoid recurrence. To evaluate the safety of and recurrence rate using this mesh, a nonrandomised prospective multicentre study was performed.

**Method:**

Fifty patients requiring surgery for parastomal hernia were enrolled. Clinical examination and CT scan prior to surgery were performed. All patients were operated on using the Parastomal Hernia Patch (BARD). Postoperative follow-up at one month and one year was scheduled to detect complications and hernia recurrence.

**Results:**

The postoperative complication rate at one month was 15/50 (30%). The parastomal hernia recurrence rate at one year was 11/50 (22%). The reoperation rate at one month was 7/50 (14%), and further 5/50 (10%) patients were reoperated on during the following eleven months.

## 1. Introduction

Parastomal hernia is a common complication in patients receiving a stoma, with a reported rate between 0 and 78% [1–3]. It may cause difficulties with stomal dressing and leakage of stomal content, as well as risk for incarceration [4, 5]. The exact incidence of hernia is difficult to determine, depending on the method of investigation, whether or not a bulge is considered a hernia, and the duration of follow-up. Most parastomal hernias manifest within a few years after the index stoma operation, but there are reports claiming hernia formation as late as 20 years after surgery [4].

There is no superior treatment for manifest parastomal hernia [1]. Several methods have been used such as stoma relocation, sutures to narrow the opening of the fascia and abdominal wall, fixation of the intestine forming the stoma to the fascia, or closure of the space lateral to the intestine. None has been shown to prevent the high rate of hernia recurrence. Mesh repair has a lower recurrence rate, 0–33%, than repair without mesh [6]. Papers reporting systematic evaluation of techniques that include sufficient numbers of patients to provide enough power for conclusive outcome and risk estimates are rare. No single technique has been shown to have superior outcome, and no randomised controlled study has been performed comparing different methods for repair of parastomal hernia. As a result, no gold standard exists with which to compare new techniques. Two principally different intraperitoneal on-lay mesh (IPOM) techniques have been developed, the Sugarbaker and the keyhole technique, the former possibly providing better results when performed with laparoscopy [7]. Furthermore, sublay mesh repair has been shown to have a considerably high recurrence rate of 7.9–14.8 [6].

Safety aspects are important when deciding on surgical repair of a benign disease with largely relative indications. In the case of parastomal hernia, very few data have been published. One study showed high morbidity with a reoperation rate of 13.2% due to postoperative complications, a high mortality rate of 6.3%, and a parastomal hernia recurrence rate of 10.8% [8].

IPOM techniques are widely used to treat incisional [9] as well as parastomal hernia. A commercially available mesh, the Parastomal Hernia Patch (BARD) (PHP), has been developed and tailored for the treatment of parastomal hernia in open and laparoscopic surgery. No systematic evaluation of the technique has been published. In order to evaluate the safety of and hernia recurrence rate using the PHP, a prospective multicentre study was conducted.

## 2. Methods

### 2.1. Study Design

The study was a single-arm nonrandomised prospective multicenter study. Data were collected prospectively in a database and patients were followed according to the protocol with individual case report forms. The study was registered at ClinicalTrials.gov identifier: NCT02233465.

### 2.2. Patients

Fifty consecutive patients with parastomal hernia requiring surgery due to leakage, problems with stomal dressing, bulging, incarceration, and other complaints related to the hernia were enrolled in the study after informed consent. Patients who did not agree to participate were operated on according to each participating department's routine.

Demographic and patient-related data, including gender, ASA, smoker or not, body mass index (BMI), presence of incisional hernia at surgery, bleeding volume during surgery, and type of stoma, were recorded.

### 2.3. Computed Tomography (CT)

The preoperative workup included clinical examination and CT of the abdomen. CT was performed in the supine position, with or without intravenous contrast fluid, at the radiology department at each participating hospital. The images were evaluated by experienced radiologists as a routine investigation. A hernia was defined as a peritoneal sac protruding through the fascia beside the stoma bowel. In all patients included, a peritoneal sac protruded, with or without content of the intestine or omentum, beside the stoma bowel.

### 2.4. Surgery

**Figure 1:**
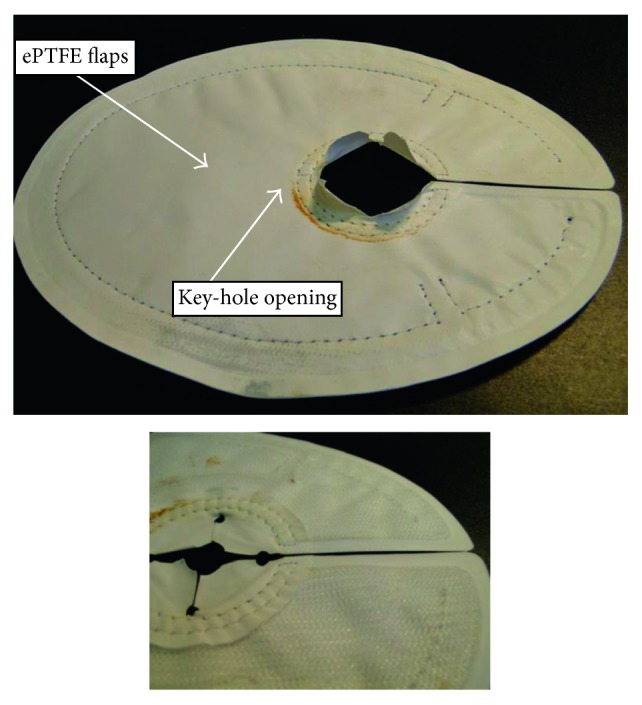
Picture of the Parastomal Hernia Patch (BARD).

The PHP consists of two layers, polypropylene and polytetrafluoroethylene (ePTFE), designed not to adhere to the abdominal content. It is manufactured in two sizes (12.5 × 15.5 cm and 15.5 × 20.5 cm), with slits in the ePTFE layer for placing tackers. These openings allow the tackers to be placed through the polypropylene layer only; by sliding the tacker device in the slit, the tackers are covered by the ePTFE layer. The mesh is designed for open and laparoscopic surgery. The shape of the mesh is oval with a keyhole opening where the intestine is allowed to pass through to the stomal orifice (Figure 1()). At surgery, the stomal opening was measured, and a mesh was placed intraperitoneally by open technique. After selecting the suitable size based on the size of the opening of the hernia and the calibre of the stoma intestine, the polypropylene component of the mesh was placed facing the abdominal wall to enable ingrowth of the mesh while the ePTFE layer faced the intestines.

After replacing the hernia from the hernia sac, the mesh was fixated to the abdominal wall with tackers and the keyhole opening was overlapped beside the intestine. No sutures were used to narrow the hernia defect. Three to four monofil sutures were used to fix the intestine to the small ePTFE flaps at the orifice of the mesh (Figure 1()). One dose of prophylactic antibiotic was given prior to surgery according to each participating hospital's routine.

Surgery at the four participating hospitals was performed by experienced colorectal surgeons with a special interest in parastomal hernia. One surgeon experienced in the PHP technique trained the participating surgeons to ensure optimised and equal technique. At each hospital, no more than one or two surgeons performed these operations. The total number of operations per individual surgeon was at least 100 major procedures each year, including parastomal hernia repair. Postoperatively, the patients were allowed to mobilise according to the hospital routine. Early complications were evaluated at one-month follow-up, and late complications and possible recurrence of parastomal hernia were assessed at one-year follow-up. Bulging was an ocular definition by the surgeon and/or the patient, but clinical examination with digital palpation in the stoma orifice during rest and Valsalva maneuver could not reveal a parastomal hernia. CT was performed after one year.

### 2.5. Statistics

Analyses of hernia recurrence and early and late complications were made using the IBM SPSS Statistics 22 software package. Comparison of hernia seen on the CT with those found at clinical examination was made using cross-tabulation.

### 2.6. Ethics

The study protocol adhered to the criteria of the Helsinki Declaration and was approved by the Ethics Committee at Umeå University, Umeå, Sweden 09-021M.

The authors did not receive any economic or other form of support from the manufacturer (BARD Company) of the prosthetic material used, and the study was initiated and conducted on an academic basis.

## 3. Results

Fifty patients, 23 male and 27 female, from four hospitals were recruited consecutively between January 2008 and January 2014. All patients had permanent stoma: 33 with colostomy, 8 with ileostomy, and 9 with urostomy. The median BMI was 26.8 (range 15.6–37.7), and the median age was 72 years (range 23–93). Patient characteristics are shown in Table 1. All included patients completed follow-up. During the study period, 31 patients (10, 9, 7, and 5, resp., at the hospitals) were operated on due to parastomal hernia and were not included in the study.

All patients underwent open elective surgery using the PHP. Median parastomal hernia defect, not including the bowel diameter, was 5.0 cm (2–10), and the smaller mesh (12.5 × 15.5 cm) was used in 35 cases while the larger (15.5 × 20.5 cm) was used in 15 cases. According to European Hernia Society classification of parastomal hernia, 23 cases were type I, 2 cases were type II, 24 cases were type III, and the remaining case was type IV [10]. All meshes were fixated with resorbable tackers (Ethicon Securestrap™ or Bard PermaSorb™) to the abdominal wall and with three to four monofil stitches to attach the ePTFE flaps to the intestine. Additional sutures were applied as single stitches to fixate the mesh to the abdominal wall. In three cases, there were concomitant incisional hernias. In two of these cases, a separate mesh was applied, and in one case, the larger PHP was positioned overlapping the midline as well. Mean duration of surgery was 110 minutes (40–377), and blood loss during surgery was 66 ml (0–750). Length of postoperative stay in hospital was 4 days (1–22).

At the one-month postoperative follow-up, complications were found in 15/50 (30%) patients. Fourteen of them were judged to have surgical complications while the fifteenth patient had pneumonia and urinary tract infection. The surgical complications comprised six wound infections, five deep infections, and three postoperative intestinal obstructions. Two of the deep infections were shown to be caused by small intestinal leakage and the other three by intra-abdominal abscesses which could be drained. 7/50 patients (14%) were reoperated on during the first postoperative month (Table 2). In three cases, the mesh was removed: two of these with incarcerated recurrent parastomal hernia and the third, mentioned earlier, with small intestinal leakage. The opening in the mesh was very tight in the two patients with incarcerated parastomal hernias.

At one-year follow-up, 11/50 (22%) patients had parastomal hernia at clinical examination and 17/50 (34%) had bulging around the stoma (Table 3). Eight of the eleven clinically found hernias also gave symptoms corresponding to hernia. Symptoms corresponding to parastomal hernia, bulging, and clinical findings were congruent in 34/50 (68%) patients. Three patients were censored after the one-month follow-up since their mesh had been removed at reoperation. CT scans were performed in 47/50 patients (94%, censored cases excluded) where parastomal hernia was seen in 7/47 (15%) patients. Omentum was found in the hernia in one patient, and in six patients, different parts of the intestine were seen in the hernia. Eight patients had protrusion of the intestine forming the stoma, as seen on CT scan. Protrusion was defined as the intestine, forming the stoma, by telescoping or sliding in the abdominal wall forming an excess of extrafascial intestine subcutaneuosly causing obstruction and/or bulging. This phenomenon was not defined as parastomal hernia. When findings at clinical assessment with hernias seen on the CT scans were compared, the results were congruent in 39/47 (83%) of the patients and incongruent in 17% (8/47) (Table 4). In six patients, clinically judged as having recurrent parastomal hernia, no hernias were found at CT. In the remaining two patients, with hernias detected at CT scan, there were no clinical signs of hernia. The need for reoperation was evaluated when examining the patients, resulting in 7/50 patients considered to gain from another operation due to the recurrence of parastomal hernia. In the group operated on with the smaller mesh, 5/35 (14%) patients were considered to need a reoperation, and the corresponding number was 2/15 (13%) in the group operated on with the larger mesh. Four of the seven patients were not reoperated on since they declined further surgery. The remaining three patients were reoperated on, and they are included in the five reoperated cases shown in Table 3. Reoperation after the one-month follow-up was performed in 5/50 cases (10%), two of these due to recurrent parastomal hernia. In total, early reoperations included, 12/50 (24%) patients had been reoperated on at the one-year follow-up. Distributions of complications according to Clavien-Dindo [11] are reported in Tables 2 and 3.

In total (one-month and one-year follow-up), four patients were reoperated on due to recurrent parastomal hernia, and in three of these cases, the opening in the mesh had become very tight, and in the fourth case, the opening had become much wider. The opening in the mesh was very tight in the two incarcerated parastomal hernias. In the case with cutoff intestine, the mesh had become tight in the opening, thereby cutting the bowel. Two cases operated on due to small bowel obstruction were shown to be caused by adhesions, and the three cases of intestinal leakage were probably caused by adhesions to the edge of the mesh. Three patients had the mesh removed at one-month follow-up. There was no additional mesh removal up to one-year follow-up. No CT scan after one year was performed in the three cases with mesh removal at one month. None of the reoperations at one-year follow-up included mesh removal.

## 4. Discussion

The recurrence rate of parastomal hernia surgery using PHP in this study must be regarded as high, taking into consideration the possibility of even later parastomal hernia recurrences. These results also bring into doubt the safety of this technique although no death had occurred within one year.

At one-year follow-up, the parastomal hernia recurrence rate was 11/50 (22%), censored cases included. At the same time, the total reoperation rate after one year was high (12/50 (24%)), and already after one month, 7/50 (14%) patients had been reoperated on. High BMI and/or comorbidities were not associated with higher recurrence or complication rate.

Urinary tract infection, pneumonia, and superficial wound infection were considered mild complications since they did not require hospitalisation. The majority of the complication, however, must be rated as serious. Within one month after the index operation, the mesh had been removed in two patients due to deep infection and intestinal obstruction. In another case with small intestinal leakage, the mesh cut off the colon resulting in peritonitis, a very serious condition. When approximately half of the patients were recruited, and one-month data were collected, a decision was made in the research group to complete inclusion to achieve robust data regarding risk for complications and hernia recurrence. One main reason for this decision was earlier studies with alternative mesh materials for parastomal hernia treatment showing similar figures regarding early complications [8].

Parastomal hernia rates vary between 0 and 78% [2]. The rationale of using IPOM when repairing parastomal hernia is to reduce recurrence risk. Surgical repair of parastomal hernia with mesh may give a lower recurrence rate than repair without mesh [2, 6]. Laparoscopic approach has been proposed as a better alternative than open technique due to less trauma to the abdominal wall. The Sugarbaker technique appears in studies to be better than the keyhole technique, with reported recurrence rates of 6.6%–12% for the Sugarbaker technique and 37% for the keyhole technique [12–14]. Follow-up times in these studies were relatively short (24–36 months). A small study proposes the double-mesh technique—the Sandwich technique—as an option for parastomal hernia repair [15]. There is no systematic previous knowledge about hernia recurrence rate or risk for complication when using PHP, and no comparison with previous data of comparable quality was available for this prospective study.

There is no well-documented method to treat parastomal hernia to be used as the gold standard for calculating power. The design of this study was intended to provide reasonable base for future power calculations. A sample multicentre cohort of 50 patients should be sufficient to define a method and is well in line with other cohorts when calculating power for larger reconstructive surgical procedures. This is the reason why the present study was designed as a controlled prospective study rather than a randomised study.

Materials including ePTFE may shrink but have the benefit of not adhering to the abdominal content [16]. Surrounding tissue is probably not robust enough to prevent shrinkage of ePTFE, despite the use of both tackers and sutures. It has been used in the treatment of incisional hernia over a considerably long period of time. Polypropylene also has shrinkage potential and may cause considerable wrinkling by fibrosis during tissue remodeling and repair. The difference between the two materials might introduce risk of exposing the polypropylene to the intestines if the ePTFE shrinks and gives rise to a larger opening in this material. Early or late enteral fistulas might be the result when exposing polypropylene to the intra-abdominal cavity. Shrinkage might give a much smaller opening, and this was considered as the explanation to the cutoff intestine in one patient. If the shrinkage results in a larger opening, this could be explained by high intra-abdominal pressure, but the true reason to a tighter or wider opening is not fully explained. Studies using other mesh materials such as polyvinylidene (PVDF) report promising results when treating parastomal hernia through short follow-up times (11–20 months) [15]. The ideal mesh material and where to place the mesh have still to be determined.

The ideal way to deal with parastomal hernia is to prevent its occurrence. No technical factor, such as site of stoma-formation or type of incision related to the construction of the stoma, has been shown to prevent hernia formation. The use of a mesh in the sublay position at the index operation has been proposed to prevent the development of a hernia [17–20]. Although results may seem promising, previous studies have been small and have not addressed the issue of late complications depending on the method of mesh implantation. One Swedish study showed similar parastomal hernia recurrence rates with and without a prophylactic mesh at the index operation [21]. The common guidelines by the National Board of Health and Welfare in Sweden stipulate prophylactic mesh at the stoma site as a field for research and, at this point, not to be used in routine practice.

At the one-year follow-up, the recurrence rate in this study was 22% by clinical examination, which must be regarded as fairly high since a parastomal hernia can develop as late as 20 years after the index operation [4]. The recurrence rate based on CT was 15%; a probable reason to this lower figure compared to the clinical findings is the difficulty to distinguish between a bulge and a hernia by clinical examination. Gurmu et al. showed interobserver reliability to be very low in clinical assessment of parastomal hernia, and they also showed CT revealing herniations not detected by clinical assessment [22]. If bulging is judged to be a parastomal hernia, the recurrence rate was 34% (Table 3). This also illustrates the difficulty in diagnosing a parastomal hernia. The different hernia recurrence rates in this study might reflect the fact that the intestine forming the stoma in some cases protrudes through the abdominal wall. This can cause bulging and be deemed a hernia at clinical examination. Protrusion may be identified by CT and is also detectable on three-dimensional (3D) ultrasonography [23]. 3D ultrasonography is a promising novel technique with the advantages of not exposing the patient to radiation and its easy accessibility [24].

Development of an effective method for treatment of parastomal hernia is a field of research that must be given priority. Laparoscopic application of the Sugarbaker technique has shown 6.6% recurrence of parastomal hernia and a complication rate of 19% [14]. The Sugarbaker technique might have a lower recurrence rate in comparison to the keyhole technique [7], and due to this, the Sugarbaker technique should be the preferred method of the two. Method of choice should have low recurrence rate and low complication risk. IPOM is widely used to treat incisional hernia with good results. The mesh technique studied in the present study using PHP does not seem to be the optimal way forward in the search for a standard technique for parastomal hernia repair, due to its high morbidity and hernia recurrence rate.

There is still no gold standard for treatment of parastomal hernia. New biological implants have been suggested as an alternative. Implantation of autologous tissue is another option. Further research in this field is urgently required.

## Figures and Tables

**Table 1:** Patient characteristics.

Patient gender	Female	27
	Male	23

Age (year)	Median (range)	72 (23–93)

BMI	Median (range)	26.8 (15.6–37.7)

ASA	ASA 1	2
	ASA 2	30
	ASA 3	17
	ASA 4	1

Smoking habits	Smoker	3
	Nonsmoker	47

Type of stoma	Colostomy	33
	Ileostomy	8
	Urostomy	9

Hernia size (cm)	Median (range)	5.5 (2–15)

BMI: body mass index, *n* = 50.

**Table 2:** One-month postoperative follow-up.

				Clavien-Dindo
Postoperative complications				
Nonsurgical	1 (2%)	Pneumonia and urinary tract infection	1	II
Surgical	14 (28%)	Wound infection	6	I
		Deep infection	5	IIIa
		Postoperative ileus	3	II
		Prolapse of the stoma	1	II

Reoperation	7 (14%)	Mesh removed due to infection and incarcerated recurrent parastomal hernia	2	IIIb
		Intestine cutoff at the level of the mesh with peritonitis, mesh removed	1	IV
		Intestinal leakage	3	IIIb
		Incision in abdominal wall due to wound infection	1	IIIb

*n* = 50.

**Table 3:** One-year follow-up.

Parastomal hernia clinically found	11/50 (22%)			
Parastomal bulging	17/50 (34%)			
CT performed	47/50	(3 patients had the mesh removed at one-month follow-up, not followed at one year)		
Parastomal hernia found by CT	7/47 (15%)	Omentum in hernia	1	
		Other part of intestine in hernia	6	
Protrusion of the stoma found by CT	8/47 (17%)			

				Clavien-Dindo

Reoperation	5/50 (10%)	Second operation for parastomal hernia	1	IIIb
		Obstruction of small intestine	2	IIIb
		Acute incarceration of parastomal hernia	1	IIIb
		Ventral hernia (not parastomal hernia)	1	IIIb

**Table 4:** Cross-table comparing findings at clinical judgement with findings at CT scan.

Clinically found hernia	CT revealing hernia		Total
	Yes	No	
Yes	5	6	11
No	2	34	36
Total	7	40	47
